# *Endonura* Cassagnau in Iran, with a key to species of the genus (Collembola, Neanuridae, Neanurinae)

**DOI:** 10.3897/zookeys.553.6009

**Published:** 2016-01-14

**Authors:** Adrian Smolis, Morteza Kahrarian, Agata Piwnik, Dariusz Skarżyński

**Affiliations:** 1 Institute of Environmental Biology, University of Wrocław, Przybyszewskiego 63-77, 51-148 Wrocław, Poland; 2 Department of Plant breeding and Agronomy, College of Agriculture, Kermanshah Branch, Islamic Azad University, Kermanshah, Iran

**Keywords:** Springtails, taxonomy, new species

## Abstract

Three new species of *Endonura* are described from Iran. *Endonura dichaeta* sp. n. can be recognized by an ogival labrum, head without chaetae O and E, chaeta D connected with tubercle Cl, tubercle Dl with five chaetae on head, absence of tubercles Di on thorax I and tubercle (Di+Di) of thorax V with 2+2 chaetae. *Endonura ceratolabralis* **sp. n.** is characterized by large body size, reduction of labral chaetotaxy, ogival labrum, head without chaeta O and fusion of tubercles Di and De on first thoracic segment. *Endonura persica* sp. n. is distinguished from its congeners by a nonogival labrum, absence of chaeta O, tubercles Dl and (L+So) with five and eight chaetae respectively and claw with inner tooth. The key to all species of the genus is given.

## Introduction

*Endonura* was established by Cassagnau (1979) as one of four subgenera within the genus *Neanura* MacGilliwray, 1893. Later, Deharveng (1982) raised it to the generic level. At present, *Endonura* is one of the largest (37 valid species) and most accurately studied genera within the subfamily Neanurinae (Dallai 1983, Deharveng 1979, 1982, Fanciulli and Dallai 2008, Pomorski and Skarżyński 2000, Pozo and Simón 1982, Smolis and Kaprus’ 2003, 2009, Smolis 2006, Smolis et al. 2007, 2011). It is mostly a Palaearctic genus and only one species, *Endonura reticulata* (Axelson, 1905), is known from the Nearctic (Alaska, Smolis et al. 2011). According to a recent definition (Smolis 2008), *Endonura* is characterized by the following characters: 0–2 ocelli, reduced mouth parts with a thin mandible and a styliform maxilla, separate tubercles Di and De on the head, the non-cross-type of chaetotaxy on the head and three or two tubercles on abdomen V. The highest species diversity is observed in Europe (32 from among the 37 known species). However, this may be a false picture because many areas of the Palaearctic have been poorly studied by collembologists. Undoubtedly, one of such regions is Central Asia, but in this case the situation is rapidly and positively changing (Arbea and Kahrarian 2015, Kahrarian 2014, Kahrarian et al. 2013, Mayvan et al. 2015, Shayanmehr et al. 2013, Smolis et al. 2012). In the present paper, three new non-European *Endonura* from the western part of Iran are described. An updated key to all species of the genus is included.

## Terminology

Terminology for the description follows that given in Deharveng (1983), Deharveng and Weiner (1984), Smolis and Deharveng (2006) and Smolis (2008).

**Abbreviations used**:

General morphology: abd. – abdomen, ant. – antenna, AOIII – sensory organ of antennal segment III, Cx – coxa, Fe – femur, Scx2 – subcoxa 2, T – tibiotarsus, th. – thorax, Tr – trochanter, VT – ventral tube.

Groups of chaetae: Ag – antegenital, An – chaetae of anal lobes, ap – apical, ca – centroapical, cm – centromedial, cp – centroposterior, d – dorsal, Fu – furcal, vc – ventrocentral, Ve or ve – ventroexternal, Vea – ventroexternoanterior, Vem – ventroexternomedial, Vep – ventroexternoposterior, Vel – ventroexternolateral, Vec – ventroexternocentral, Vei – ventroexternointernal, Vi or vi – ventrointernal, Vl – ventrolateral.

Tubercles: Af – antenno–frontal, Cl – clypeal, De – dorsoexternal, Di – dorsointernal, Dl – dorsolateral, L – lateral, Oc – ocular, So – subocular.

Types of chaetae: Ml – long macrochaeta, Mc – short macrochaeta, Mcc – very short macrochaeta, me – mesochaeta, mi – microchaeta, ms – s–microchaeta or microsensillum, S or s – chaeta s, bs – border s–chaeta on ant. IV, miA – microchaetae on ant. IV, iv – ordinary chaetae on ventral ant. IV, or – organite of ant. IV, brs – border s–chaeta on ant. IV, i – ordinary chaeta on ant. IV, mou – cylindrical s–chaetae on ant. IV („soies mousses”), x – labial papilla x, L’ – ordinary lateral chaeta on abd. V, B4, B5 – ordinary chaetae on tibiotarsi.

## Materials and methods

The specimens were cleared in Nesbitt’s fluid, subsequently mounted on slides in Swan’s medium and observed using a phase contrast microscope Nikon E600. Photographs were made using a camera Nikon D5100 mounted on a microscope mentioned above. Photographs were stacked using Helicon Focus 6.2.2. and prepared for publication using Adobe Photoshop CS3. Material is deposited in the Department of Invertebrate Biology, Evolution and Conservation, Institute of Environmental Biology, University of Wrocław, Poland.

## Taxonomy

### Endonura dichaeta sp. n.

Taxon classificationAnimaliaCollembolaNeanuridae

http://zoobank.org/4CBE64B2-069B-4254-AF20-43D26E6CFE10

Figs 1–4 , Table 1

#### Type material.

Holotype: adult female on slide, Iran, Osmanevand area, near Golestan village (N33°55', E47°06', 1241 m a.s.l.), litter in oak forest, 13.XII.2013, leg. M. Kahrarian. Paratypes: female, two males and two juveniles on slides, same data as holotype.

#### Other material.

Two females and male on slide, Iran, Osmanevand area, near Chelkooshk village (N34°03', E47°12', 1516 m a.s.l.), litter in oak forest, 31.I.2014, leg. M. Kahrarian; three juveniles on slide, Iran, Paveh county, near Shabankereh village (N34°52.978', E46°30.760', 1632 m a.s.l.), litter in oak forest, 20.I.2014, leg. M. Kahrarian; two females and juvenile, Iran, Kermanshah county, near Chahar zebra-e-oliya village (N34°13', E46°40', 1592 m a.s.l.), litter in oak forest, 24.I.2014, leg. M. Kahrarian.

#### Etymology.

The species name refers to rare feature within the genus - only two chaetae Di on each side of tubercle (Di+Di) of abdomen V.

#### Diagnosis.

Habitus typical of the genus *Endonura*. Dorsal tubercles present and well developed, except tubercles Di on th. I. 2+2 unpigmented eyes. Buccal cone long, labrum ogival. Head with chaetae A, B, C, D, F and G. Chaetae O and E absent. Tubercles Cl and Af separate. Tubercle Cl with chaetae D. Tubercles Dl and (L+So) on head with five and eight chaetae respectively. Tubercles De on th. II and III with three and four chaetae respectively. Tubercles L on abd. III and IV with three and six chaetae respectively. Abd. IV and V with eight and three tubercles respectively. Claw without inner tooth. Tibiotarsi with chaetae B4 and B5 short.

#### Description.

**Figures 1–4.**
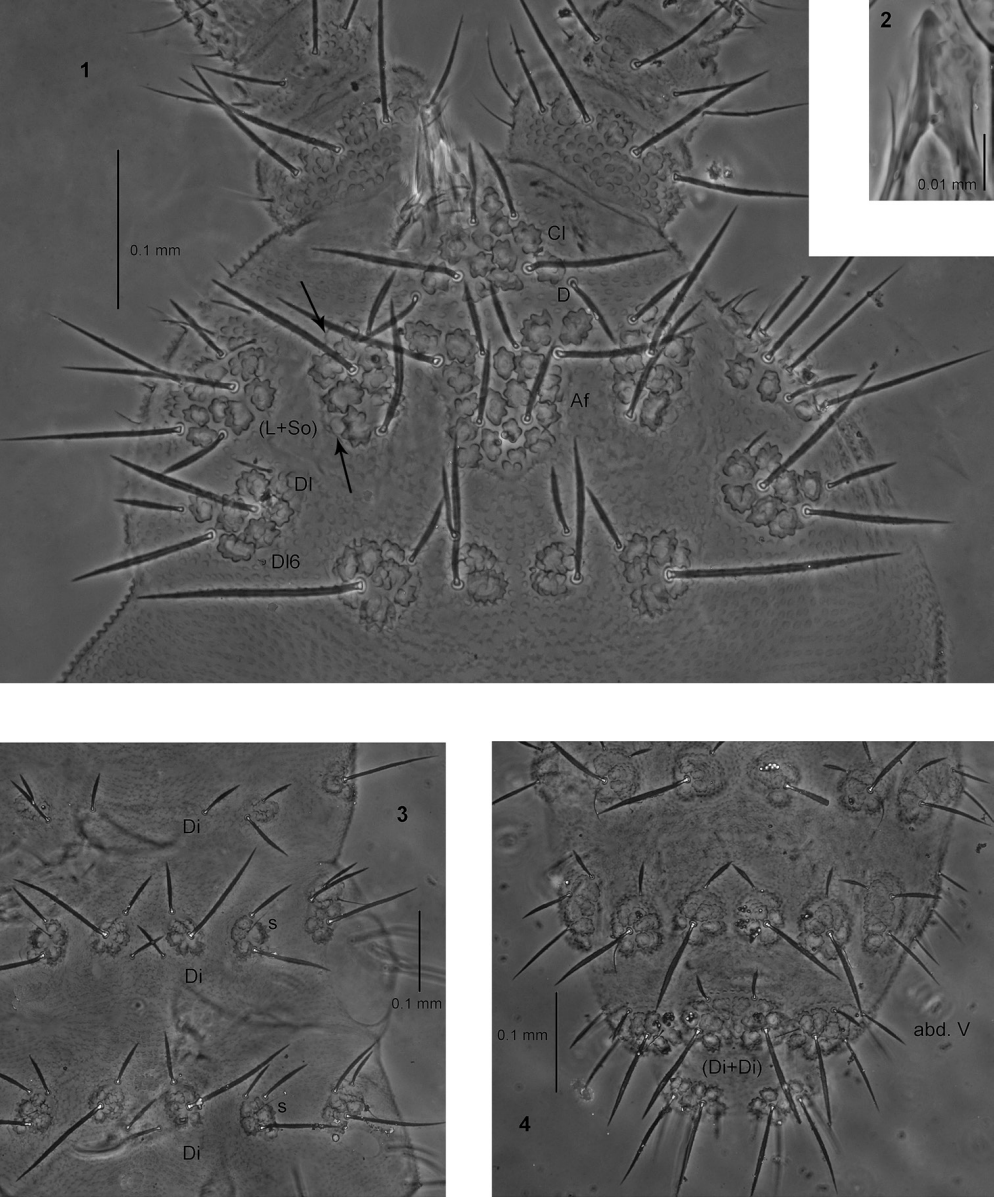
*Endonura dichaeta* sp. n.: **1** head (holotype), dorsal and lateral chaetotaxy **2** ventral sclerification of labrum **3** dorsal chaetotaxy of thorax **4** dorsal chaetotaxy of abdomen III–VI. Arrows indicate the position of eyes.

Habitus typical of the genus. Body length (without antennae): 0.75–1.55 mm (holotype 1.30 mm). Colour of the body white. 2+2 medium unpigmented eyes (Fig. 1).

Types of dorsal ordinary chaetae. Macrochaetae Ml relatively long, strongly thickened, almost cylindrical, arc-like or straight, narrowly sheathed, feebly serrated, apically pointed (Figs 1, 3–4); macrochaetae Mc and Mcc thickened, straight and pointed; mesochaetae and microchaetae short, thin, feebly serrated and pointed.

Head. Labrum ogival, with ventral sclerifications as in Fig. 2. Labrum chaetotaxy 2/2, 4. Labium with four basal, three distal and four lateral chaetae, papillae x absent. Maxilla styliform, mandible thin tridentate. Chaetotaxy of antennae as in Table 1c. Apical vesicle distinct, trilobed. S–chaetae of ant.IV long and moderately thickened. Chaetotaxy of head as in Table 1a, b, and Fig. 1. Chaeta D connected with tubercle Cl. Tubercle Af on head longer than tubercles Oc. Tubercle Dl with five chaetae, chaeta Dl3 absent, chaeta Dl6 as minute microchaeta and hard to detect (Fig. 1). Tubercle (L+So) with eight chaetae, chaetae So2 and L3 absent, chaeta So6 as Mc (Fig. 1). Elementary tubercles BE and CD present. Chaeta A shorter than B.

**Table 1a.** Chaetotaxy of *Endonura dichaeta* sp. n.: Cephalic chaetotaxy–dorsal side.

Tubercle	Number of chaetae	Types of chaetae	Names of chaetae
Cl	6	Ml Mc	F D, G
Af	6	Ml Mc	A B, C
Oc	3	Ml Mc	Ocm, Ocp Oca
Di	2	Ml Mc	Di1 Di2
De	2	Ml Mc	De1 De2
Dl	5	Ml Mc Mcc mi	Dl1, Dl5 Dl4 Dl2 Dl6
(L+So)	8	Ml Mc me	L1, L4, So1 L2, So6 So3–5

**Table 1b.** Chaetotaxy of *Endonura dichaeta* sp. n.: Cephalic chaetotaxy–ventral side.

Group	Number of chaetae
Vi	6
Vea	3
Vem	3
Vep	4
labium	11, 0x

**Table 1c.** Chaetotaxy of *Endonura dichaeta* sp. n.: Chaetotaxy of antennae.

Segment, Group	Number of chaetae	Segment, Group	Number of chaetae adult
I	7	IV ap	or, 8 S, i, 12 mou, 6 brs, 2 iv
II	11		
III ve	5 sensilla AO III		
	5		8 bs, 5 miA
vc	4	ca	2 bs, 3 miA
vi	4	cm	3 bs, 1 miA
d	5	cp	8 miA, 1 brs

Thorax, abdomen, legs. Body s–chaeta thin and smooth, shorter than nearby macrochaetae (Figs 3, 4). Chaetotaxy of th. and abd. as in Table 1d and in Figs 3, 4. Tubercles Di on th.I not differentiated. Chaetae De3 on th. III and abd. I–III as Mcc. Chaetae De2 on th. II–III and De3 on th. III connected with tubercle De. Chaetae De3 on abd. I–III connected with tubercle De (Fig. 4). The line of chaetae De1–chaeta s not perpendicular to the dorsomedian line on abd I–III. Furca rudimentary with 2–4 microchaetae. Tubercles Di on abd. V fused, with chaetae Di2 as Mc or Mcc, chaetae Di3 absent (Fig. 4). Chaetae L’ and Vl on abd. V present. IV abd. with 2+2 chaetae Ag. No cryptopygy. Chaetotaxy of legs as in Table 1d.

**Table 1d.** Chaetotaxy of *Endonura dichaeta* sp. n.: Postcephalic chaetotaxy.

	Di	Terga De	Dl	L	Scx2	Cx	Legs Tr	Fe	T
th. I	1	2	1	-	0	3	6	13	19
th. II	3	2+s	3+s+ms	3	2	7	6	12	19
th. III	3	3+s	3+s	3	2	8	6	11	18
							Sterna		
abd. I	2	3+s	2	3	VT: 4				
abd. II	2	3+s	2	3	Ve: 4–5	Ve1 -	present		
abd. III	2	3+s	2	3	Vel:4–5			Fu:5–6me	2–4mi
abd. IV	2	2+s	3	6	Vel: 4	Vec: 2	Vei: 2	Vl: 4	
abd. V	(2+2)	5+s			Ag: 2			Vl: 1	L’: 1
abd. VI		7			Ve:13–14			An: 2mi	

#### Remarks.

In general appearance (shape of dorsal chaetae, chaetotaxy of central area of head and dorsal side of thorax and abdomen, complete absence of pigmentation and absence of cryptopygy), *Endonura dichaeta* sp. n. strongly resembles *Endonura tartaginenis* Deharveng, 1980 described from Corsica. Nevertheless, both taxa differ in some essential characters, important from taxonomic point of view: presence/absence of chaetae E on head (*dichaeta* sp. n. absent, *tartaginenis* present), number of chaetae Dl on head (*dichaeta* sp. n. five, *tartaginenis* six), number of chaetae (L+So) (*dichaeta* sp. n. eight, *tartaginenis* nine), presence/absence of elementary tubercle EE on head (*dichaeta* sp. n. absent, *tartaginenis* present), presence/absence of tubercles Di on the first thoracic segment (*dichaeta* sp. n. absent, *tartaginenis* present), number of chaetae Di on abd. V (*dichaeta* sp. n. 2+2, *tartaginenis* 3+3) and presence/absence of tooth on claw (*dichaeta* sp. n. absent, *tartaginenis* present). In addition, the new species is characterized by only 2+2 antegenital chaetae (*tartaginenis* 3+3) and ogival labrum (unknown in *tartaginenis*), characters rarely observed within the genus.

### Endonura ceratolabralis sp. n.

Taxon classificationAnimaliaCollembolaNeanuridae

http://zoobank.org/FC09DDF3-EB60-416D-B31C-A290A4E812D5

Figs 5–9 , Table 2

#### Type material.

Holotype: adult female on slide, Iran, Osmanevand area, near Markhor village (N33°53', E47°05', 1389 m a.s.l.), litter in oak forest, 13.XII.2013, leg. M. Kahrarian. Paratypes: 3 females on slide, same data as holotype.

#### Other material.

Three females on slide, Iran, Osmanevand area, near Ghader marz village (N34°01.030', E47°12.415', 1682 m a.s.l.), litter in oak forest, 31.I.2014, leg. M. Kahrarian.

#### Etymology.

The species name refers to sharp labral apex which looks like a horn (“cera” in latin).

#### Diagnosis.

Habitus typical of the genus *Endonura*. Dorsal tubercles present and well developed. 2+2 eyes darkly pigmented. Buccal cone long. Head with chaetae A, B, C, D, E, F and G. Chaeta O absent. Tubercles Cl and Af separate. Tubercles Dl and (L+So) on head with six and nine chaetae respectively. Tuberles Di and De on th. I fused. Tubercles De on th. II and III with three and four chaetae respectively. Tubercles L on abd. III and IV with three and 6–7 chaetae respectively. Abd. IV and V with eight and three tubercles respectively. Claw without inner tooth. Tibiotarsi with chaetae B4 and B5 short.

#### Description.

**Figures 5–9.**
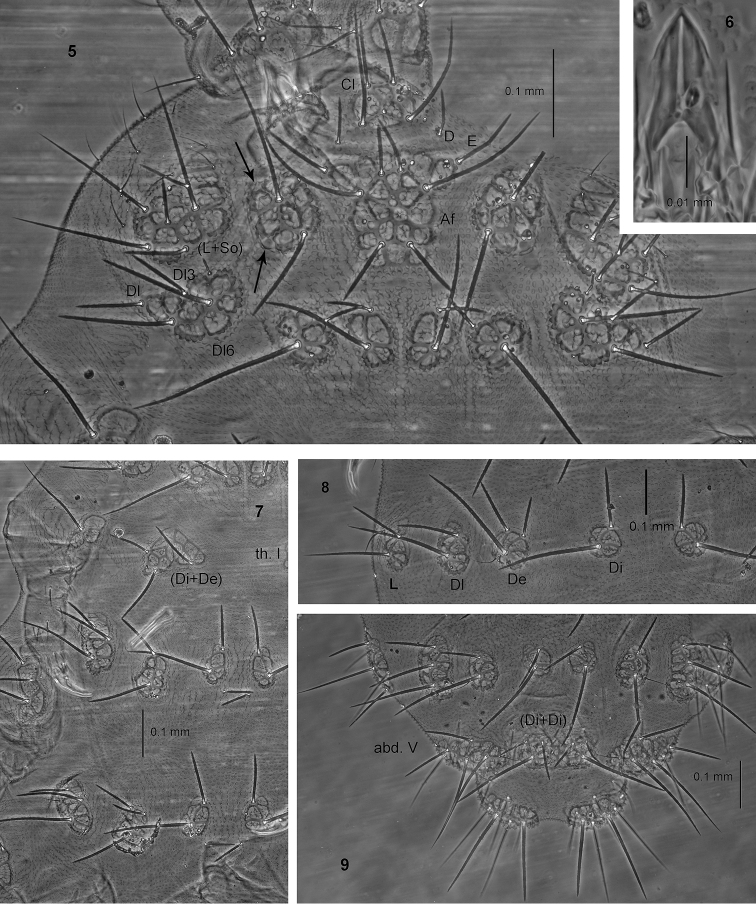
*Endonura ceratolabralis* sp. n.: **5** head (holotype), dorsal and lateral chaetotaxy **6** ventral sclerification of labrum **7** dorsal chaetotaxy of thorax **8** dorsal chaetotaxy of abd. II **9** dorsal chaetotaxy of abdomen IV–VI. Arrows indicate the position of eyes.

Habitus typical of the genus. Body length (without antennae): 2.25–2.55 mm (holotype: 2.55 mm). Colour of the body bluish grey. 2+2 medium dark-pigmented eyes (Fig. 5).

Types of dorsal ordinary chaetae. Macrochaetae Ml thickened, relatively long, arc-like or straight, narrowly sheathed, feebly serrated, apically pointed or rarely rounded (Figs 5, 7–9); macrochaetae Mc and Mcc thickened, straight, pointed or apically rounded; mesochaetae and microchaetae short, thin and pointed.

Head. Buccal cone very long. Labrum ogival, with ventral sclerifications as in Fig. 6. Labrum chaetotaxy 0/2, 2. Labium with four basal, three distal and four lateral chaetae, papillae x absent. Maxilla styliform, mandible thin with two basal and two apical teeth. Chaetotaxy of antennae as in Table 2c. Apical vesicle distinct, trilobed. S–chaetae of ant.IV of medium length and moderately thickened. Chaetotaxy of head as in Table 2a, b, and Fig. 5. Tubercles Cl and Af separate. Tubercle Af on head longer than tubercles Oc. Chaeta O absent. Chaeta D free. Tubercle Dl with six chaetae, chaeta Dl3 present. Tubercle (L+So) with nine chaetae, chaeta So2 absent and chaeta So3 as Mc (Fig. 5). Elementary tubercles BE and CD present. Chaeta A shorter than B.

**Table 2a.** Chaetotaxy of *Endonura ceratolabralis* sp. n.: Cephalic chaetotaxy–dorsal side.

Tubercle	Number of chaetae	Types of chaetae	Names of chaetae
Cl	4	Ml Mc	F G
Af	10	Ml Mc	A B, C, D, E
Oc	3	Ml me	Ocm, Ocp Oca
Di	2	Ml Mc	Di1 Di2
De	2	Ml Mc	De1 De2
Dl	6	Ml Mc mi	Dl1, Dl5 Dl2, Dl3, Dl4 Dl6
(L+So)	9	Ml Mc me	L1, L4, So1 L2, L3, So3 So4–6

**Table 2b.** Chaetotaxy of *Endonura ceratolabralis* sp. n.: Cephalic chaetotaxy–ventral side.

**Group**	**Number of chaetae**
Vi	6
Vea	3–4
Vem	3
Vep	4
Labium	11, 0x

**Table 2c.** Chaetotaxy of *Endonura ceratolabralis* sp. n.: Chaetotaxy of antennae.

**Segment, Group**	**Number of chaetae**	**Segment, Group**	**Number of chaetae adult**
I	7	IV ap	or, 8 S, i, 12 mou, 6 brs, 2 iv
II	12–14		
III ve	5 sensilla AO III		
	5		8 bs, 5 miA
vc	4	ca	2 bs, 3 miA
vi	4	cm	3 bs, 1 miA
d	5	cp	8 miA, 1 brs

Thorax, abdomen, legs. Body s–chaetae thin and smooth, shorter than nearby macrochaetae (Figs 7–9). Chaetotaxy of th. and abd. as in Table 2d and in Figs 7–9. Tubercles Di on th.I differentiated and fused with De (Fig. 7). Dorsal side of th. and abd. without free chaetae De. The line of chaetae De1–chaeta s perpendicular to the dorsomedian line on abd I–III. Furca rudimentary with two or without microchaetae. Tubercles Di on abd. V fused, with chaetae Di2 and Di3 as Mc (Fig. 9). Chaetae L’ and Vl on abd. V present. No cryptopygy. Chaetotaxy of legs as in Table 2d.

**Table 2d.** Chaetotaxy of *Endonura ceratolabralis* sp. n.: Postcephalic chaetotaxwy.

	Di	Terga De	Dl	L	Scx2	Cx	Legs Tr	Fe	T
th. I	1	2	1	-	0	3	6	13	19
th. II	3	2+s	3+s+ms	3	2	7	6	12	19
th. III	3	3+s	3+s	3	2	8	6	11	18
							Sterna		
abd. I	2	3+s	2	3	VT: 4				
abd. II	2	3+s	2	3	Ve: 5–6	Ve1 -	Present		
abd. III	2	3+s	2	3	Vel:4–5			Fu:3–6me	0–2mi
abd. IV	2	2+s	3	6–7	Vel: 4	Vec: 2	Vei: 2	Vl: 4	
abd. V	(3+3)	5+s			Ag: 3			Vl: 1	L’: 1
abd. VI		7			Ve: 13–14			An: 2mi	

#### Remarks.

Because of the very characteristic long and pointed labrum, *Endonura ceratolabralis* sp. n. seems to be most similar to *Endonura cretensis* (Ellis, 1976) (Crete) and *Endonura gracilirostris* Smolis et al. 2007 (Crimea). Nevertheless, the new species can be easily distinguished from these two taxa by the following combination of characters: maximum length of the body without antennae (*ceratolabralis* sp. n. 2.55 mm; *gracilirostris* 1.45 mm; *cretensis* 0.8 mm), labral formula (*ceratolabralis* sp. n. 0/2, 2; *gracilirostris* 0/2, 4; *cretensis* 2/2, 4), presence/absence of chaeta O on head (*ceratolabralis* sp. n. absent, in others present), number of chaetae Dl on head (*cretensis* three, in others six), number of chaetae (L+So) on head (*cretensis* seven, in others nine), fusion/separation of tubercles Di and de on th. I (*gracilirostris* separate, in others fused), number of ordinary chaetae De on th. III (*cretensis* two, others three), presence/absence of free chaetae on thorax (*ceratolabralis* sp. n. absent, in others present) and number of chaetae Di on abd. V (*cretensis* 1-2, others three).

### Endonura persica sp. n.

Taxon classificationAnimaliaCollembolaNeanuridae

http://zoobank.org/9CFE5947-62CC-4A3E-ABF7-5B84EA69A21A

**Figures 10–13.**
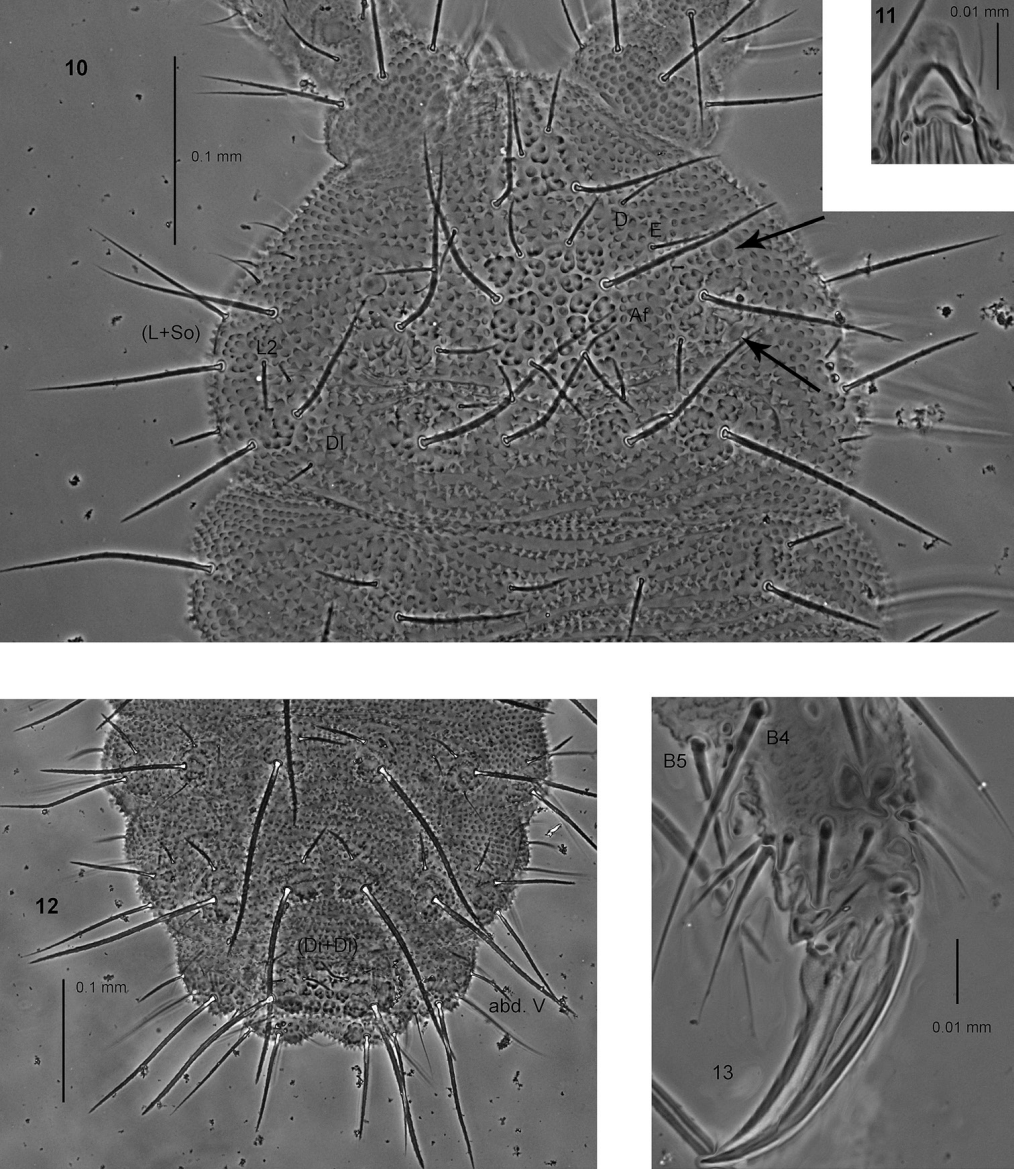
*Endonura persica* sp. n.: **10** head and th. I, dorsal and lateral chaetotaxy **11** ventral sclerification of labrum **12** dorsal chaetotaxy of abdomen III–VI (holotype) **13** tibiotarsi and claw of leg III. Arrows indicate the position of eyes.

Figs 10–13 , Table 3

#### Type material.

Holotype: adult female on slide, Iran, Kermanshah area, near Ghaleh shahin village (N34°25.590', E05°12.415', 566 m a.s.l.), litter in willow shrubs, 7.IV.2014, leg. M. Kahrarian. Paratypes: two females, four males and four juveniles on slides, same data as holotype.

#### Other material.

Female on slide, Iran, Kermanshah Province, Halashi County, near Sarfiroozabad village (N34°02', E47°10', 1624 m a.s.l.), litter in oak forest, 15.II.2014, leg. M. Kahrarian; female and male on slide, Iran, Osmanevand area, near Sarjoob village (N33°56', E47°08', 1240 m a.s.l.), litter in oak forest, 13.XII.2013, leg. M. Kahrarian.

#### Etymology.

The species name refers to the historic name of Iran, Persia.

#### Diagnosis.

Habitus typical of the genus *Endonura*. Dorsal tubercles present and generally well developed, only tubercles Di on th. I weakly differentiated. 2+2 large dark-pigmented eyes. Buccal cone rather short. Head with chaetae A, B, C, D, E , F and G. Chaeta O absent. Tubercles Cl and Af separate. Tubercles Dl and (L+So) on head with five and eight chaetae respectively. Tubercles De on th. II and III with three and four chaetae respectively. Tubercles L on abd. III and IV with four and 6–7 chaetae respectively. Abd. IV and V with eight and three tubercles respectively. Claw with inner tooth. Tibiotarsi with chaetae B4 and B5 long.

#### Description.

Habitus typical of the genus. Body length (without antennae): 0.75–1.90 mm (holotype: 1.10 mm). Colour of the body bluish grey. 2+2 large dark pigmented eyes (Fig. 10).

Types of dorsal ordinary chaetae. Macrochaetae Ml thickened, relatively long, arc–like or straight, narrowly sheathed, feebly serrated, apically rounded or rarely pointed (Figs 10, 12); macrochaetae Mc and Mcc thickened, straight and not pointed; mesochaetae and microchaetae short, thin and pointed.

Head. Buccal cone short. Labrum rounded, with ventral sclerifications as in Fig. 11. Labrum chaetotaxy 4/2, 4. Labium with four basal, three distal and four lateral chaetae, papillae x absent. Maxilla styliform, mandible thin with two basal and two apical teeth. Chaetotaxy of antennae as in Table 3c. Apical vesicle distinct trilobed. S–chaetae of ant.IV of medium length and moderately thickened. Chaetotaxy of head as in Table 3a, b, and Fig. 10. Chaetae D and E free. Tubercles Cl and Af separate. Tubercle Af on head longer than tubercles Oc. Tubercle Dl with five chaetae, chaeta Dl3 absent. Tubercle (L+So) with eight chaetae, chaetae So2 and L3 absent (Fig. 10). Elementary tubercle BE absent. Chaeta A shorter than B.

**Table 3a.** Chaetotaxy of *Endonura persica* sp. n.: Cephalic chaetotaxy–dorsal side.

Tubercle	Number of chaetae	Types of chaetae	Names of chaetae
Cl	4	Ml Mc	F G
Af	10	Ml Mc Mc or Mcc	B A, C, E D
Oc	3	Ml Mc mi	Ocm Ocp Oca
Di	2	Ml Mcc	Di1 Di2
De	2	Ml Mcc	De1 De2
Dl	5	Ml Mc or Mcc	Dl1, Dl5 Dl2, Dl4, Dl6
(L+So)	8	Ml Mc me or mi	L1, L4, So1 L2 So3–6

**Table 3b.** Chaetotaxy of *Endonura persica* sp. n.: Cephalic chaetotaxy–ventral side.

Group	Number of chaetae
Vi	6
Vea	3–4
Vem	3
Vep	4
labium	11, 0×

**Table 3c.** Chaetotaxy of *Endonura persica* sp. n.: Chaetotaxy of antennae.

Segment, Group	Number of chaetae	Segment, Group	Number of chaetaev adult
I	7	IV ap	or, 8 S, i, 12 mou, 6 brs, 2 iv
II	12–14		
III ve	5 sensilla AO III		
	5		8 bs, 5 miA
vc	4	ca	2 bs, 3 miA
vi	4	cm	3 bs, 1 miA
d	5	cp	8 miA, 1 brs

Thorax, abdomen, legs. Body s–chaetae fine and smooth, distinctly shorter than nearby macrochaetae (Fig. 12). Chaetotaxy of th. and abd. as in Table 3d and in Figs 10, 12. Tubercles Di on th.I differentiated or not. Chaetae De2 on th. II–III and De3 on th. III free. Chaetae De3 on abd. I–III free (Fig. 12). The line of chaetae De1–chaeta s parallel to the dorsomedian line on abd. I–III. Furca rudimentary without microchaetae. Tubercles Di on abd. V fused, with chaetae Di2 as Mcc and Di3 as mi (Fig. 12). Chaetae Vl on abd. V present. Cryptopygy slightly developed. Chaetotaxy of legs as in Table 3d. Tibiotarsi with rather long chaetae B4 and B5. Claw with inner tooth (Fig. 13).

**Table 3d.** Chaetotaxy of *Endonura persica* sp. n.: Postcephalic chaetotaxy.

	Di	Terga De	Dl	L	Scx2	Cx	Legs Tr	Fe	T
th. I	1	2	1	-	0	3	6	13	19
th. II	3	2+s	3+s+ms	3	2	7	6	12	19
th. III	3	3+s	3+s	3	2	8	6	11	18
							Sterna		
abd. I	2	3+s	2	3	VT: 4				
abd. II	2	3+s	2	3	Ve: 5–6	Ve1 -	present		
abd. III	2	3+s	2	4	Vel: 5			Fu:5–10me	0 mi
abd. IV	2	2+s	3	6–7	Vel: 4	Vec: 2	Vei: 2	Vl: 4	
abd. V	(3+3)	8+s			Ag: 3			Vl: 1	L’: 1
abd. VI		7			Ve: 13–14			An: 2mi	

#### Remarks.

In general appearance and presence of inner tooth on claw, characters rarely observed within the genus, *Endonura persica* sp. n. strongly resembles to *Endonura dentifera* Smolis et al. 2007 (described from Crimea). However, the new species can be reliably separated from Crimean species with the following characters: number of chaetae Dl on head (*persica* sp. n. five, *dentifera* six), number of chaetae (L+So) on head (*persica* sp. n. eight, *dentifera* ten), presence/absence of tubercles Di on the first thoracic segment (*persica* sp. n. present, *dentifera* absent) and number of chaetae L of abd. IV (*persica* sp. n. 6–7 chaetae, *dentifera* 8–9).

### Key to the genus *Endonura*

In 1982, Deharveng, in his PhD thesis, elevated *Endonura* to the generic level and prepared a key to the genus that comprised 23 species. Nowadays, including the taxa described herein, the genus contains 40 members and is the second largest of the tribe Neanurini, after *Deutonura* Cassagnau, 1979. Moreover, after the publication of Deharveng’s paper (date), a few species were redescribed and one taxon was synonymised (Smolis and Kaprus’ 2003, Smolis 2008, Smolis et al. 2007, 2011). Considering these facts, the preparation of an updated key to all species of the genus seemed to be highly recommended.

**Table:** 

1	Head with fusion of tubercles Af and Cl	**2**
–	Head with separation of tubercles Af and Cl	**7**
2.	Chaeta O on head present	**3**
	Chaeta O on head absent	**4**
3	Tubercles Di on th. I present and fused with De, tubercle (Di+Dl+L) on abd. V with nine chaetae	***Endonura poinsotae* Deharveng, 1980** (France, Corsica)
–	Tubercles Di on th. I absent, tubercle (Di+Dl+L) on abd. V with seven chaetae	***Endonura ichnusae* Dallai, 1983** (Italy, Sardinia)
4	Tubercles De on abd. I–III with four chaetae	**5**
–	Tubercles De on abd. I–III with three chaetae	***Endonura granulata* (Cassagnau & Delamare Deboutteville, 1955)** (Lebanon)
5	Tubercles Di and De on th. I fused, cryptopygy strongly developed	***Endonura gladiirostra* Smolis & Kaprus’, 2003** (Israel)
–	Tubercles Di and De on th. I separate, cryptopygy absent or weakly developed	**6**
6	Chaeta E on head present, Tubercle Dl on head with four chaetae	***Endonura tyrrhenica* Dallai, 1983** (Italy, Sardinia)
–	Chaeta E on head absent, Tubercle Dl on head with six chaetae	***Endonura pejai* Deharveng, 1980** (France, Corsica)
7	Tubercle Af on head equal or shorter than tubercles Oc	**8**
–	Tubercle Af on head longer than tubercles Oc	**9**
8	Labrum with ventral sclerifications ogival and without prelabral chaetae	***Endonura gracilirostris*Smolis et al., 2007** (Crimea, Moldova)
–	Labrum with ventral sclerifications nonogival and with prelabral chaetae	***Endonura taurica* (Stach, 1951)** (Crimea)
9	Chaeta O on head present	**10**
–	Chaeta O on head absent	**26**
10	Eyes completely absent	**11**
–	Eyes present	**12**
11	Tubercles Di on th. I present	***Endonura arbasensis* Deharveng, 1979** (France, Spain)
–	Tubercles Di on th. I absent	***Endonura caeca* (Gisin, 1963)** (Bosnia and Herzegovina)
12	Anterior eye present and located outside tubercle Oc	***Endonura asiatica*Smolis et al., 2011** (Kyrgyzstan)
–	Anterior eye present or absent, if present located within tubercle Oc	**13**
13	Anterior eye present	**14**
–	anterior eye absent	***Endonura immaculata* Deharveng, 1980** (France, Corsica)
14	Claw with inner tooth, tibiotarsi with long chaetae B4 and B5	**15**
–	Claw without tooth, tibiotarsi with short chaetae B4 and B5	**16**
15	Tubercle Dl on head with three chaetae, tubercles Di on th. II–III with two chaetae	***Endonura tetrophtalma* (Stach, 1929)** (Hungary)
	Tubercle Dl on head with five chaetae, tubercles Di on th. II–III with three chaetae	***Endonura lusatica* (Dunger, 1966)** (Germany, Poland, Ukraine)
16	Chaeta E on head absent	**17**
–	Chaeta E on head present	**18**
17	Tubercle Cl on head with chaetae D, elementary tubercle DF present	***Endonura colorata* (Gama, 1964)** (Portugal)
–	Tubercle Cl on head without chaetae D, elementary tubercle DF absent	***Endonura cantabrica* (Deharveng, 1979)** (Spain)
18	Tubercle Dl on head with six chaetae	**19**
–	Tubercle Dl on head with less number of chaetae	**25**
19	Tubercles Di on head present	**20**
–	Tubercles Di on head absent	***Endonura dalensi* Deharveng, 1979** (Andorra, France, Spain, Italy)
20	Body white	**21**
–	Body blue or bluish–grey	**22**
21.	Tubercle (L+So) on head with nine chaetae, macrochaetae thin and pointed	***Endonura deharvengi* Cassagnau & Péja, 1979** (Greece)
–	Tubercle (L+So) on head with eight chaetae, macrochaetae thickened and blunt	***Endonura levantica*Smolis et al., 2011** (Israel)
22	Tubercle De on th. III with two ordinary chaetae	***Endonura gladiolifer* (Cassagnau, 1954)** (Algeria, Spain)
–	Tubercle De on th. III with three ordinary chaetae	**23**
23	Tubercle Cl on head with chaetae D, furca rudimentary with microchaetae	***Endonura alavensis* Pozo & Simon, 1982** (Spain)
–	Tubercle Cl on head without chaetae D, furca rudimentary without microchaetae	**24**
24	Tubercle (L+So) on head with nine chaetae, free chaeta L on abd. IV present	***Endonura quadriseta* Cassagnau & Péja, 1979** (Greece, Turkey, Crimea)
–	Tubercle (L+So) on head with ten chaetae, free chaeta L on abd. IV absent	***Endonura reticulata* (Axelson, 1905)** (Finland; Russia; Sweden; United States, Alaska)
25	Tubercle Dl on head with four chaetae, tubercles Di and De on th. I separate	***Endonura occidentalis* (Deharveng, 1979)** (Spain)
–	Tubercle Dl on head with three chaetae, tubercles Di and De on th. I fused	***Endonura cretensis* (Ellis, 1976)** (Greece, Israel)
26	Cryptopygy strong and complete, tubercles of abd. VI invisible in dorsal view	***Endonura ludovicae* (Denis, 1948)** (France, Corsica)
–	Cryptopygy absent or weak, tubercles of abd. VI well or partially visible in dorsal view	**27**
27	Body bluish–grey	**28**
–	Body white	**30**
28	Claw with inner tooth, labrum chaetotaxy 4/2, 4	**29**
–	Claw without inner tooth, labrum chaetotaxy 0/2, 2	***Endonura ceratolabralis* sp. n.** (Iran)
29	Tubercle Dl on head with five chaetae, tubercles Di on th. I present	***Endonura persica* sp. n.** (Iran)
–	Tubercle Dl on head with six chaetae, tubercles Di on th. I absent	***Endonura dentifera*Smolis et al., 2007** (Crimea)
30	Chaeta C on head absent	**31**
–	Chaeta C on head present	**33**
31	Macrochaetae Di1 on abd. V distinctly thickened and club–like	***Endonura baculifer* Deharveng, 1979** (Portugal)
–	Macrochaetae Di1 on abd. V slightly thickened and cylindrical	**32**
32	Eyes present, tubercles Di on th. I present	***Endonura transcaucasica* (Stach, 1951)** (Georgia)
–	Eyes absent, tubercles Di on th. I absent	***Endonura carpatica* Smolis, 2006** (Poland)
33	Tubercle Cl on head with chaetae D, elementary tubercle DF present	**34**
–	Tubercle Cl on head without chaetae D, elementary tubercle DF absent	**35**
34	Chaeta E on head present, tubercle Dl on head with six chaetae	***Endonura tartaginenis* Deharveng, 1980** (France, Corsica)
–	Chaeta E on head absent, tubercle Dl on head with five chaetae	***Endonura dichaeta* sp. n.** (Iran)
35	Chaeta E on head present	***Endonura urotuberculata* Pomorski & Skarżyński, 2000** (Bulgaria)
–	Chaeta E on head absent	**36**
36	Chaeta L4 on head free, eyes absent or present unpigmented	**37**
–	Chaeta L4 within tubercle (L+So), eyes present and pigmented	**39**
37	Abd. V with two tubercles	***Endonura incolorata* (Stach, 1951)** (Poland, Ukraine, Romania)
–	Abd. V with three tubercles	**38**
38	Abd. IV with eight tubercles, macrochaetae Ml relatively short	***Endonura tatricola* (Stach, 1951)** (Poland, Slovakia)
–	Abd. IV with five tubercles, macrochaetae Ml long	***Endonura dudichi* (Loksa, 1967)** (Hungary, Poland, Slovakia)
39	Tubercle Dl on head with six chaetae, tubercle L on abd. III with three chaetae	***Endonura centaurea* Cassagnau & Péja, 1979** (Greece)
–	Tubercle Dl on head with five chaetae, tubercle L on abd. III with four chaetae	***Endonura saleri* Fanciulli & Dallai, 2008** (Italy)

## Discussion

Considering the data presented here and those obtained from the literature (Mayvan et al. 2015, Shayanmehr et al. 2013, Smolis et al. 2012), Neanurinae fauna of Iran comprises ten species and seven genera: *Bilobella aurantiaca* (Caroli, 1912), *Cryptonura persica* Smolis et al., 2012, *Cryptonura maxima* Smolis et al., 2012, *Deutonura decolorata* (Gama & Gisin, 1964) (Gisin 1964), *Endonura ceratolabralis* sp. n., *Endonura dichaeta* sp. n., *Endonura persica*, sp. n., *Neanura muscorum* (Templeton, 1835), *Persanura hyrcanica* Mayvan et al. 2015, *Thaumanura echinata* (Kos, 1940). It should be noted, however, that until now only the western part of Iran has been roughly studied. Although future research may change the present picture of the subfamily diversity in the studied country and region, some preliminary conclusions can be drawn. The first is related to the higher systematic pattern and composition of Neanurinae of Iran. This fauna consists almost exclusively of members of the tribe Neanurini, the most diverse and dominant among Neanurinae in the western Palaearctic. To date, none of the Lobellini and Paranurini genera have been found in Iran, although they are numerous and widely distributed in south, south-east and east Asia. The second conclusion seems to be more expected, *Endonura* species from Iran resemble those known from south-east Europe. It suggests their close affinity and the historical connection between these faunas. The third conclusion sheds light on the distribution and the history of this genus. Most *Endonura* species were recorded from Mediterranean and temperate zones of Europe, where they live predominantly in forests. It is worth saying that the greatest diversity of the genus through the continent is more or less correlated to the areas of land that have never been subjected to glaciations. Till now, the occurrence of only a few species is documented outside Europe, especially in the Middle East (Smolis and Kaprus 2003, 2009; Smolis et al. 2011). The recent and present discoveries of *Endonura* species in Kyrgyzstan (Smolis et al. 2011) and Iran significantly expand the list of species and also our knowledge on the genus. Undoubtedly, diverse forest habitats of the coastal and montane regions of Iran and adjacent countries hide a rich fauna of Neanurinae. We therefore hope that a more comprehensive study in the future will allow us to present a better picture of the distribution of *Endonura* in Iran and the near East.

## Supplementary Material

XML Treatment for Endonura dichaeta

XML Treatment for Endonura ceratolabralis

XML Treatment for Endonura persica
